# Detection of human papilloma virus in breast cancer biopsies using PCR and immunohistochemistry at the santa rosa hospital during 2019

**DOI:** 10.17843/rpmesp.2022.394.11069

**Published:** 2022-12-15

**Authors:** Enrique Mamani- Zapana, Meyling Vilcapoma-Diaz, Nazario Ortiz-Muchotrigo

**Affiliations:** 1 Laboratory of Clinical Molecular Virology, School of Biological Sciences, Universidad Nacional Mayor de San Marcos, Lima, Peru. Universidad Nacional Mayor de San Marcos Laboratory of Clinical Molecular Virology School of Biological Sciences Universidad Nacional Mayor de San Marcos Lima Peru; 2 Ecovirus Research Group, School of Biological Sciences, Universidad Nacional Mayor de San Marcos, Lima, Peru. Universidad Nacional Mayor de San Marcos Ecovirus Research Group School of Biological Sciences Universidad Nacional Mayor de San Marcos Lima Peru; Department of Clinical Pathology and Anatomic Pathology, Hospital Santa Rosa, Lima, Peru. Department of Clinical Pathology and Anatomic Pathology Hospital Santa Rosa Lima Peru

**Keywords:** Human Papillomavirus type 16 and 18, breast cancer, biopsy, qPCR, IHC

## Abstract

The aim of this study was to determine the presence of Human Papillomavirus (HPV) type 16 and 18 in biopsies of paraffin-embedded breast tissue from patients with clinically diagnosed breast cancer. 32 paraffin-embedded breast cancer biopsies were analyzed in order to detect HPV DNA by real-time PCR, the primers were directed at the E6 gene. The histological type, histological grade and overexpression of C-erB2 and Ki-67 were evaluated by immunohistochemistry. 84.38% (27) of the samples were positive for HPV, 25% (8) were positive for HPV-16 and 59.38% (19) were positive for HPV-18. Mixed infection was found in 15.63% (5) of the samples. Overexpression of C-erbB2 and Ki-67 was seen in 6.25% (2) of the samples positive for HPV-16 and in 15.63% (5) samples positive for HPV-18. HPV-16 and HPV-18 DNA was detected in the biopsy samples analyzed by real-time PCR.

## INTRODUCTION

Breast cancer (BCa) is one of the most complex disease ^1^ that occurs gradually ^2^ and constitutes a worldwide public health concern that mainly affects women. Internationally, breast cancer is considered the fifth type of cancer with the highest number of deaths, with approximately 685,000 deaths ^3^. In Peru, breast cancer is the most frequent cancer in women between 40 and 69 years of age ^4^, who are usually diagnosed in the last stages ^5^.

This neoplasm is related to modifiable factors (parity, age at first pregnancy, breastfeeding, and lifestyle) and non-modifiable factors (genetic mutations, family history, sex, age, race, ethnicity, dense breasts, age at menarche and late menopause) ^6^. However, it is difficult to determine a single risk factor that causes breast cancer, since some women with several risk factors do not develop cancer and some women who are not exposed to risk factors do develop cancer ^7^.

In recent decades, HPV has been the risk factor most frequently associated with BCa, mainly due to reports finding the genetic material of this virus in women diagnosed with BCa. In 2008, Khan *et al*. examined the presence, genotype, and viral load of HPV in 124 Japanese patients diagnosed with mammary carcinoma ^8^, detecting the HPV genome in 26 patients, in whom HPV-16 (92%) was the most frequent. In 2015, Lawson *et al.* detected the presence of HPV in 50 specimens from a total of 855 samples with mammary carcinoma, of which 20 were high-risk (HPV-18, HPV-16, HPV-52 and HPV-113) and 30 were low-risk ^9^. In 2018, Chumpitaz confirmed the existence of HPV-18 and HPV-16 in breast tumor tissue in Peruvian women ^10^.

Due to previous reports and the increase in breast cancer cases in Peru, the present study sought to determine the presence of HPV type 16 and type 18 in breast carcinoma biopsies from patients who visited the Hospital Santa Rosa during 2019.

**KEY MESSAGES**

Motivation for the study: there are few studies about high-risk Human Papillomavirus in patients with breast cancer, which is currently the most recurrent neoplasm in Peru.

Main findings: greater presence of Human Papillomavirus was evidenced in infiltrating ductal carcinoma and grade III samples. In addition, real-time polymerase chain reaction showed greater diagnostic accuracy than immunohistochemistry.

Implications: a better understanding of the presence of Human Papillomavirus and its possible relationship with breast cancer will contribute to improve preventive measures for this disease.

## THE STUDY

### Design and place of study

Descriptive-retrospective study carried out at the Santa Rosa Hospital (H.S.R.) and at the Universidad Nacional Mayor de San Marcos.

### Variables

The independent variables were divided into two groups: social (age) and clinical (type of breast carcinoma, histological grade, and overexpression of C-erbB2 and Ki-67 receptors). The dependent variable was the presence of HPV-16 and HPV-18. We used the statistical package IBM SPSS Statistics for Windows, version 25 (IBM Corp). The dependent variable was expressed by sector graphs and the independent variables by histogram graphs. In addition, we constructed a comparative graph of the qPCR and IHC techniques using the XLSTAT statistical software. Absolute and relative frequencies were used.

### Sample collection

We analyzed 32 paraffin-embedded breast tissue biopsy samples from female patients older than 30 years (age range: 32-93 years) with BCa who visited the H.S.R. during January-April 2019. Participants were selected consecutively (routinely), and samples from all patients with BCa during the study period were included. Each sample was obtained in triplicate from 2-micrometer-thick histological sections deposited on poly-L-lysine slides.

### Extraction and evaluation of DNA quality

The NucleoSpin ® DNA FFPE XS kit was used, following the manufacturer’s instructions ^11^. DNA concentration and quality were measured with a DeNovix spectrophotometer, model DS-11FX+, using 2uL per sample; readings were taken at 260 NM and A260 / A280 ratio, respectively.

### Real-time PCR

HPV was detected by qPCR following the methodology described by Frega *et al*. ^10^ and modified for the present study. The probe and primers were targeted to the E6 gene as described by Schmitz *et al*. ^11^ (Table 1). The SensiFAST™ Probe No-ROX Kit (Bioline, Meridian Bioscience) was used ^12^. The qPCR required a volume of 20 µL for each reaction: 10 µL of Taq polymerase (2X SensiFast Probe No-ROX Mix), 0.8 µL of primer (HPV-16/18 For and HPV-16 /18Rev), 0.4 µL of TaqMan probe (HPV-16 /18Probe) ,7 µL of ultrapure water, and 1 µL of viral DNA were used. Cycling conditions were optimized as shown in Table 2. Positive controls (HPV-16 and HPV-18 cervical positive sample) and a negative control were included in each reaction.

**Table 1:** Sequence of the primers and TaqMan probe for the detection of the E6 gene of HPV-16 and HPV-18.

Oligonucleotides	Sequence (5’-3’)	qPCR product size
Oligonucleotides for HPV-16		128 bp
HPV-16 For	GAACCGAAACCGGTTAGTATAA	
HPV-16 Rev	ATGTATAGTTGTTTGCAGCTCTGT	
HPV-16 p	6HEX-CATTTTATGCACCAAAAGAGAACTGCAATGTTTC-BHQ1	
Oligonucleotides for HPV-18		
HPV-18 For	GGACCGAAAACGGTGTATATAA	124 bp
HPV-18 Rev	CAGTGAAGTGTTCAGTTCGGT	
HPV-18 p	TAMRA-ATGTGAGAAACACACCACAATACTATGGCGCG-BHQ2	

**Table 2:** Cycling program for HPV qPCR

Step	Temperature	Time	No. of cycles
Initial activation	95 °C	15 min	1
Denaturation	94 °C	5 s	45
Hybridization	50 °C	20 s	
Extension	60 °C	40 s	

### Immunohistochemistry (IHC)

Tissue sections with a thickness of 2 microns were deposited on poly-L-lysine slides. The following kits were used: BIO SB Mouse/Rabbit Immunodetector HRP/DAB, BIO SB Tinto HPV-16 Mouse Antibody, clone CAMVIR-1, Santa Cruz Biotechnology HPV-18 Mouse Antibody, clone E6. C-erB2 and Ki-67 were used to measure overexpression in tumor tissues.

### Ethical aspects

This research was approved by the Research Ethics Committee of Hospital Santa Rosa.

## FINDINGS

### Real-time PCR

Of the 32 samples from patients with BCa, 84.38% (27) were positive for HPV, of which 25% (8) corresponded to HPV-16 and 59.38% (19) to HPV-18. Mixed infection was found in 15.63% (5) of the samples.

### Relation of the independent variables with HPV-16 and HPV-18 PCR

The age range was 32 to 93 years, with a median of 59 years. Of the total HPV-16 positive cases (8), 50% (4) were younger than 59 years and the other 50% (4) were older than 59 years. Regarding histological grade, 75% (6/8) corresponded to grade III and 25% (2/8) to grade II. Regarding the type of BCa, 87% (7/8) of the samples presented infiltrating ductal carcinoma and 13% (1/8) intraductal carcinoma.

Of the HPV-18 positive cases (19), 42% (8/19) were younger than 59 years and 58% (11/19) were older than 59 years. Regarding the histological grade, 58% (11/19) corresponded to grade III, 37% (7/19) to grade II and 5% (1/19) to grade I. The distribution according to the type of BCa was 95% (18/19) for infiltrating ductal carcinoma and 5% (1/19) for intracystic carcinoma.

### Immunohistochemistry

**Figure 1:**
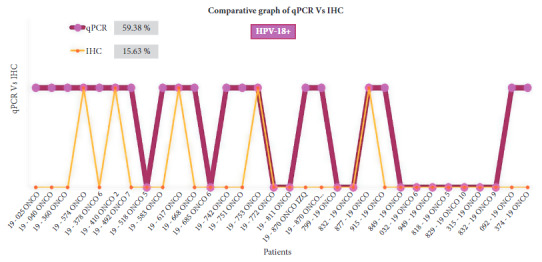
Comparative graph between the results of qPCR and IHC techniques for HPV-18. Of the 19 samples positive for HPV- 18 by qPCR, 5 samples overexpressing ki-67 and C-erbB2 receptors were positive by the two techniques: 19 - 574 ONCO (sample 4), 19 - 410 ONCO 2 (sample 6), 19 - 617 ONCO (sample 10), 19 - 753 ONCO (sample 15) and 877 -19 ONCO (sample 22)

**Figure 2:**
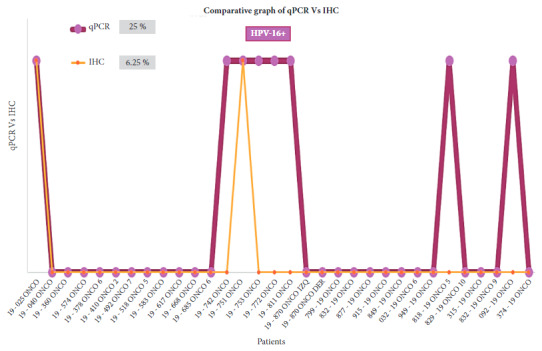
Comparative graph between the results of qPCR and IHC techniques for HPV-16. Of the 8 samples positive for HPV-16 by qPCR, 2 samples overexpressing ki-67 and C-erbB2 receptors were positive by the two techniques: 19-025 ONCO (sample 1) and 19-751 ONCO (sample 14).

Of the 32 samples analyzed for HPV-16 and HPV-18, only 6.25% (2) and 15.63% (5) showed overexpression of the immunohistochemical markers C-erbB2 and Ki-67, respectively (Figures 1 and 2). HPV-16 and HPV-18 positive samples with overexpression of C-erbB2 and Ki-67 presented the infiltrating ductal carcinoma type and grade III.

## DISCUSSION

The present study demonstrated the presence of HPV-16 and HPV-18 in BCa samples, by using the E6 gene as a detection target, which remains preserved and is not lost during viral integration as occurs with the L1 gene ^13^; this is probably the reason why previous studies did not report HPV in BCa samples.

Our results coincide with previous reports that used primers targeting the E6 gene. In 2009, Cantú de León *et al*. ^14^ determined the prevalence of HPV DNA, collecting 51 BCa cases at the National Cancer Institute of Mexico; 29.4% ^15^ were positive for HPV, 66.6% ^10^ of these were positive for HPV-16, 20% ^3^ were positive for HPV-18 and 13.4% ^2^ were positive for both. Similarly, in 2017 Ngamkhan *et al*. ^15^ evaluated the prevalence of HPV in 700 samples of Thai women with BCa by analyzing the statistical correlation between HPV infection and sociodemographic and histopathological characteristics; the researchers found that patients with benign breast tumor lesions had an average age of 41.76 and those with breast carcinoma samples averaged 52.73 years of age, they also detected HPV DNA in 25 genotypes in 700 samples with breast tumors, which accounted for 3.57% of the total. HPV-16 was the most prevalent, followed by HPV-33 and HPV-18. In our study, we found 25% (8/32) of HPV-16 and 59.38% (19/32) of HPV-18. This indicates that the prevalence of HPV infection varies between different geographical areas and would be related to non-modifiable factors such as genetic factors and ethnicity, among others, which give rise to the competition of the virus for population niches and results in some types being more prevalent. Of the total number of genotypes found, 15.63% (5) corresponded to mixed infections, related to grade II and grade III of BCa and also to the type of infiltrating ductal carcinoma. This allows us to infer that in addition to viral competition between HPV types in the same tissue, the infection persists and breast lesions progress. 

The age of the women positive for HPV-16 and HPV-18 varied, so it was not possible to determine the relationship between age and the HPV type. However, Kroupis *et al*. ^22^, after evaluating the presence of HPV in cases of BCa in 107 samples, found that 21 samples were positive for high-risk HPV, 14 of them being younger than the rest of the patients. On the other hand, Khan *et al*. ^8^ found no significance between age and the presence of HPV in BCa, the ages of the patients ranged from 23 to 90 years and the median was 55 years; of the 15 cases that were younger than 40 years, 3 were positive for HPV; of the 37 cases that had ages between 40 and 49, 9 were positive for HPV; of the 27 patients with ages between 50 and 59 years, 5 had the HPV viral genome and of the 45 cases that had ages between 60 and 93, 9 were positive for HPV. Therefore, in order to establish a possible relationship between age and the presence of HPV, it is necessary to evaluate a larger number of samples so the prevalence of this virus can be analyzed. The results of the aforementioned studies coincide with the National Plan for the Prevention and Control of Breast Cancer in Peru 2017-2021, which reports that breast cancer is more frequent in Peruvian women between 40 and 69 years of age ^23^.

The overexpression of C-erbB2 and Ki-67 receptors was determined by using IHC on breast samples. Several studies have used these markers and labeled them as specific for cancer cells with a high association with high-risk HPV. The greater the expression of Ki-67, the greater the increase in C-erbB2 expression and, therefore, the greater the aggressiveness of the cancer ^16^. This would explain the greater overexpression of C-erbB2 and Ki-67 in high-grade BCa samples and would be the reason why greater overexpression of these receptors was found in infiltrating ductal carcinoma samples. On the other hand, HER-2 proteins are found on the surface of breast cells, both normal and cancerous cells ^17^. HER-2 is overexpressed by up to 30% in breast cancers ^18^. The Ki-67 protein is found in the nucleus of the human cell and expresses cell proliferation in both normal and malignant tissue, reaching maximum expression levels during mitosis ^19^. Currently, since the main characteristic of cancer is uncontrolled cell proliferation, the Ki-67 proliferative index is being used more frequently to evaluate and monitor cancer as a prognostic indicator ^18^ ^,^ ^19^. By using IHC, we found overexpression of these immunomarkers in women with BCa, positive for HPV-18 in 15.63% and for HPV-16% in 6.25%. Of the 19 samples that tested positive for HPV-18 by qPCR, 5 samples that overexpressed ki-67 and C-erbB2 receptors were positive by both techniques; and of the 8 samples that tested positive for HPV-16 by qPCR, 2 that overexpressed ki-67 and C-erbB2 receptors were positive by both techniques. This would demonstrate the high diagnostic accuracy of the qPCR technique, which would allow us to consider it as a complementary test to IHC, to possibly contribute to early BCa diagnosis. 

Our results are consistent with what Woods *et al.* described in 2015; they found that coexpression of E6 and C-erbB2 resulted in cells expressing higher levels of C-erbB2, thus concluding that there is probably an influence of the E6 oncoprotein of HPV-16 on HER-2 (C-erbB2) gene overexpression ^20^. However, there are studies that indicate the opposite. In 2015, Delgado analyzed the relationship between HPV and the molecular marker Ki-67 in 275 breast cancer samples, finding no statistical significance between the presence of HPV and Ki-67 ^21^. However, Kroupis *et al*. examined the presence of HPV in breast cancer tissues by PCR; 17 samples out of 107 were positive for HPV and statistical significance was found between HPV and Ki-67 ^22^. Therefore, it can be concluded that it is not possible to determine whether or not the overexpression of C-erbB2 and Ki-67 is related to the presence of HPV when working with few samples.

One of the limitations of this study is that the prevalence of HPV-16 and HPV-18 in breast cancer and their correlation with the immunohistochemical markers C-erbB2 and Ki-67 were not determined. On the other hand, one of the strengths of the study is that we analyzed clinical and social variables that have not been found in other studies at the national level. Likewise, we considered the different factors that influence DNA quality, so we worked with a specialized extraction kit for paraffinized breast tissue samples, standardized qPCR and worked with amplicons of less than 200 bp, which guarantee high diagnostic accuracy.

In conclusion, the presence of HPV-16 and HPV-18 was confirmed in breast carcinoma biopsies of Peruvian patients, with a higher presence of HPV in infiltrating ductal carcinoma and grade III samples. Likewise, our results have allowed us to confirm that, in addition to the presence of HPV, other intrinsic or extrinsic factors that act synergistically and predispose to the development of cancer in humans are required.
