# Supplementary material for: Detection of human papilloma virus in breast cancer biopsies using PCR and immunohistochemistry at the santa rosa hospital during 2019
Source: Rev Peru Med Exp Salud Publica. 2022 Dec 15;39(4):450–5. doi: 10.17843/rpmesp.2022.394.11069 (PMC11397775; doi:10.17843/rpmesp.2022.394.11069)
Supplement: Translation

# Translation: Deteccion del virus del papiloma humano en biopsias de cancer de mama mediante PCR e inmunohistoquimica en el Hospital Santa Rosa durante el 2019

**Authors:** Enrique Mamani- Zapana, Meyling Vilcapoma-Diaz, Nazario Ortiz-Muchotrigo

**Affiliations:** 1 Laboratorio de Virología Clínica Molecular, Facultad de Ciencias Biológicas, Universidad Nacional Mayor de San Marcos, Lima, Perú.; 2 Grupo de Investigación Ecovirus, Facultad de Ciencias Biológicas, Universidad Nacional Mayor de San Marcos, Lima, Perú.; 3 Departamento de Patología clínica y Anatomía Patológica, Hospital Santa Rosa, Lima, Perú.

**Keywords:** Virus del Papiloma Humano tipo 16 y 18, cáncer de mama, biopsia, qPCR, IHQ

## Abstract

El objetivo de este estudio fue determinar la presencia del Virus Papiloma Humano (VPH) tipo 16 y 18 en biopsias de tejido mamario parafinado de pacientes con diagnóstico clínico de cáncer de mama. Se analizaron 32 biopsias de cáncer de mama embebidas en parafina para detectar el ADN de VPH mediante PCR en tiempo real, los iniciadores estuvieron dirigidos al gen E6. Se evaluaron el tipo histológico, grado histológico y la sobreexpresión de C-erB2 y Ki-67 mediante inmunohistoquímica. El 84,38% (27) fueron positivos para VPH, el 25% (8) fueron positivos para VPH-16 y el 59,38% (19) para VPH-18. El 15,63% (5) de las muestras presentaron infección mixta. Se evidenció la sobrexpresión de C-erbB2 y Ki-67 en 6,25% (2) de las muestras positivas para VPH-16 y 15,63% (5) de las muestras positivas para VPH-18. Se detectó ADN de VPH-16 y VPH-18 en las muestras de biopsias analizadas mediante PCR en tiempo real.

## INTRODUCCIÓN

El cáncer de mama (CaMa) es una de las patologías más complejas ^1^ ^) ^que se da de forma gradual ^2^ y constituye un problema mundial de salud pública que afecta principalmente a las mujeres. A nivel internacional, el cáncer de mama es considerado el quinto tipo de cáncer con mayor número de fallecimientos, con aproximadamente 685 000 defunciones^ (^ ^3^. En el Perú, el CaMa es la neoplasia más frecuente en mujeres de 40 a 69 años ^4^, quienes son diagnosticadas en las últimas etapas del cáncer ^5^.

Esta neoplasia está relacionada con factores modificables (paridad, edad del primer embarazo, lactancia materna y estilo de vida) y no modificables (mutaciones genéticas, antecedentes familiares, sexo, edad, raza, origen étnico, mamas densas, edad de la menarquía y menopausia tardía) ^6^. Sin embargo, es difícil determinar un único factor de riesgo que cause el CaMa, ya que ciertas mujeres con varios factores de riesgo no desarrollan cáncer y algunas que no están expuestas a los factores de riesgo si lo desarrollan ^7^.

En las últimas décadas, el factor de riesgo asociado con más frecuencia al CaMa es el VPH, debido principalmente a la aparición de reportes que muestran la presencia del material genético de este virus en mujeres diagnosticadas con CaMa. En el 2008, Khan *et al*. examinaron la presencia, genotipo y carga viral del VPH en 124 pacientes japonesas diagnosticadas con carcinoma mamario ^8^, detectando el genoma de VPH en 26 pacientes, en quienes el más frecuente fue VPH-16 (92%). En el 2015, Lawson *et al*. demostraron la presencia del VPH en 50 ejemplares de un total de 855 muestras con CaMa, de las cuales 20 fueron de alto riesgo (VPH -18, VPH -16, VPH -52 y VPH -113) y 30 de bajo riesgo ^9^. En el año 2018, Chumpitaz confirmó la existencia de VPH -18 y VPH -16 en tejido tumoral de mama en mujeres peruanas ^10^.

Debido a los reportes previos y el incremento en los casos de cáncer de mama en el Perú, el presente estudio buscó determinar la presencia del VPH tipo 16 y tipo 18 en biopsias de carcinoma mamario de pacientes atendidas en el Hospital Santa Rosa durante el año 2019.

**MENSAJES CLAVE**

Motivación para realizar el estudio: Existen pocos estudios acerca del Virus del Papiloma Humano (VPH) de alto riesgo en pacientes con cáncer de mama (CaMa), la cual es actualmente la neoplasia más recurrente en Perú.

Principales hallazgos: Se evidenció mayor presencia del VPH en las biopsias de carcinoma ductal infiltrante y grado III, además, se demostró que la reacción en cadena de la polimerasa en tiempo real tuvo mayor precisión diagnóstica que la inmunohistoquímica.

Implicancias: Un mejor entendimiento de la presencia de VPH y su posible relación con CaMa contribuirán en las medidas preventivas para esta enfermedad.

## EL ESTUDIO

### Diseño y lugar de estudio

Estudio descriptivo-retrospectivo desarrollado en el Hospital Santa Rosa (H.S.R.) y en la Universidad Nacional Mayor de San Marcos.

### Variables

Las variables independientes se dividieron en dos grupos: social (edad) y clínicos (tipo de carcinoma mamario, grado histológico y sobreexpresión de los receptores C-erbB2 y Ki-67). La variable dependiente fue la presencia de VPH-16 y VPH-18. Se utilizó el paquete estadístico IBM SPSS Statistics for Windows, versión 25 (IBM Corp). La variable dependiente se expresó por gráfico de sectores y las variables independientes, por gráficos de histogramas. Además, se realizó un gráfico comparativo de las técnicas qPCR e IHQ, para ello se empleó el Software estadístico XLSTAT. Se utilizaron frecuencias absolutas y relativas.

### Recolección de muestras

Se evaluaron 32 muestras de biopsias de tejido mamario parafinado de pacientes mujeres mayores de 30 años (rango de edad: 32-93 años) con CaMa atendidas en el H.S.R. durante el periodo enero-abril del 2019. Las participantes fueron seleccionadas de manera consecutiva (rutinaria), y se incluyeron las muestras de todas las pacientes con CaMa durante el periodo de estudio. Cada muestra se obtuvo por triplicado de cortes histológicos de 2 micras de espesor depositadas en láminas de polilisina.

### Extracción y evaluación de la calidad del DNA

Se utilizó el kit NucleoSpin ® DNA FFPE XS, siguiendo las indicaciones del fabricante ^11^. Se midió la concentración y calidad del ADN con un espectrofotómetro marca DeNovix, modelo DS-11FX+, se utilizó 2uL por muestra y se procedió a las lecturas en 260 NM y relación A260 / A280, respectivamente.

### PCR en tiempo real

La detección del VPH se realizó mediante la qPCR siguiendo la metodología descrita por Frega *et al.* ^10^ ^) ^y modificada para el presente estudio. La sonda e iniciadores estuvieron dirigidos al gen E6, según lo descrito por Schmitz *et al.* ^11^ (Tabla 1). Se empleó el Kit SensiFAST™ Probe No-ROX Kit (Bioline, Meridian Bioscience) ^12^. La qPCR requirió un volumen de 20 µL por cada reacción: se empleó 10 µL de la Taq polimerasa (2X SensiFast Probe No-ROX Mix), 0.8 µL de iniciador (VPH-16/18 For y VPH-16 /18Rev), 0.4 µL de sonda TaqMan (VPH-16 /18Probe) ,7 µL de agua ultrapura, y 1 µL de ADN viral. Se optimizó las condiciones de ciclaje según se muestra en la Tabla 2. En cada reacción se incluyeron los controles positivos (muestra positiva para VPH-16 y VPH-18 de cérvix) y el control negativo.

**Tabla 1:** Secuencia de los primers y sonda TaqMan para la detección del gen E6 del HPV-16 y HPV-18.

Oligonucleótidos	Secuencia (5’-3’)	Tamaño del producto de la qPCR
Oligonucleótidos para HPV-16		128 bp
VPH-16 For	GAACCGAAACCGGTTAGTATAA	
VPH-16 Rev	ATGTATAGTTGTTTGCAGCTCTGT	
VPH-16 p	6HEX-CATTTTATGCACCAAAAGAGAACTGCAATGTTTC-BHQ1	
Oligonucleótidos para HPV-18		
VPH-18 For	GGACCGAAAACGGTGTATATAA	124 bp
VPH-18 Rev	CAGTGAAGTGTTCAGTTCGGT	
VPH-18 p	TAMRA-ATGTGAGAAACACACCACAATACTATGGCGCG-BHQ2	

**Tabla 2:** Programa de ciclaje para la qPCR de VPH.

Ciclaje			
Paso	Temperatura	Tiempo	Nº de ciclos
Activación inicial	95 *ºC*	15 min	1
Denaturación	94 *ºC*	5 s	45
Hibridación	50 *ºC*	20 s	
Extensión	60 *ºC*	40 s	

### Inmunohistoquímica (IHQ)

Secciones de tejidos de 2 micras de espesor fueron depositadas en láminas de polilisina. Se emplearon los kits: BIO SB Mouse/Rabbit Inmunodetector HRP/DAB, BIO SB Tinto HPV-16 Mouse Antibody, clona CAMVIR-1, Santa Cruz Biotechnology HPV-18 Mouse Antibody, clona E6. Se utilizó C-erB2 y Ki-67 para medir la sobreexpresión en los tejidos tumorales. 

### Aspectos éticos

Esta investigación fue aprobada por el Comité de Ética en investigación del Hospital Santa Rosa.

## HALLAZGOS

### PCR en tiempo real

De las 32 muestras de pacientes con CaMa, el 84,38% (27) fueron positivos para VPH, de las cuales, el 25% (8) correspondió a VPH-16 y el 59,38% (19) a VPH-18. El 15,63% (5) de las muestras presentaron infección mixta.

### Relación de las variables independientes con la PCR de VPH-16 y VPH-18

El rango de la edad fue 32 a 93 años, con una mediana de 59 años. Del total de casos positivos para VPH-16 (8), el 50% (4) fueron menores de 59 años y el otro 50% (4) fueron mayores de 59 años. Con respecto al grado histológico, el 75% (6/8) correspondieron al grado III y el 25% (2/8) al grado II. Con respecto al tipo de CaMa, el 87% (7/8) presentaron carcinoma ductal infiltrante y el 13% (1/8) carcinoma intraductal. 

De los casos positivos para VPH-18 (19), el 42% (8/19) fueron menores de 59 años y 58% (11/19) fueron mayores 59 años. Con respecto al grado histológico, el 58% (11/19) correspondió al grado III, 37% (7/19) al grado II y 5% (1/19) al grado I. La distribución según el tipo de CaMa fue 95% (18/19) para el carcinoma ductal infiltrante y 5% (1/19) para el carcinoma intraquístico. 

### Inmunohistoquímica

Con respecto a VPH-16 y VPH-18, se visualizó que, de las 32 muestras analizadas, solo el 6,25% ^2^ y el 15,63% ^5^ manifestaron la sobrexpresión de los marcadores inmunohistoquímicos C-erbB2 y Ki-67, respectivamente (Figuras 1 y 2). Las muestras positivas para VPH-16 y VPH-18 con expresión exagerada de C-erbB2 y Ki-67 pertenecían al tipo de carcinoma ductal infiltrante y al grado III.

**Figura 1:** Gráfica comparativa entre los resultados de las técnicas qPCR e IHQ para VPH-18. De las 19 muestras positivas para VPH-18 por qPCR, 5 muestras que sobreexpresaron los receptores ki-67 y C-erbB2 coincidieron con las dos técnicas: 19 - 574 ONCO (muestra 4), 19 - 410 ONCO 2 (muestra 6), 19 - 617 ONCO (muestra 10), 19 - 753 ONCO (muestra 15) y 877 -19 ONCO (muestra 22).

**Figura 2:** Gráfica comparativa entre los resultados de las técnicas qPCR e IHQ para VPH-16. De las 8 muestras positivas para VPH-16 por qPCR, 2 muestras que sobreexpresaron los receptores ki-67 y C-erbB2 coincidieron con las dos técnicas: 19-025 ONCO (muestra 1) y 19-751 ONCO (muestra 14).

## DISCUSIÓN

En el presente estudio se demostró la presencia de VPH -16 y VPH-18 en muestras con CaMa, utilizando como blanco de detección al gen E6, el cual se mantiene conservado y no se pierde durante la integración viral como ocurre con el gen L1 ^13^; este probablemente es el motivo por el cual estudios previos no reportaron VPH en muestras con CaMa.

Los resultados obtenidos concuerdan con reportes previos que emplearon iniciadores dirigidos al gen E6. En el 2009, Cantú de León *et al*. ^14^ ^) ^determinaron la prevalencia de DNA de VPH, recopilando 51 casos con CaMa en el Instituto Nacional de Cancerología de México; el 29,4% ^15^ ^) ^fueron positivos para VPH, 66,6% ^10^ ^) ^de estos fueron positivos para VPH-16, 20% ^3^ fueron positivos para VPH-18 y el 13,4%^ (^ ^2^ ^) ^fueron positivos para ambos. De la misma manera, en el 2017 Ngamkhan *et al.* ^15^ investigaron la prevalencia del VPH en 700 muestras de mujeres tailandesas con CaMa analizando la correlación estadística entre la infección por VPH, parámetros sociodemográficos e histopatológicos; los investigadores encontraron que las pacientes con lesiones tumorales benignas de mama tenían una edad promedio de 41,76 y aquellas con muestras con carcinoma mamario promediaron 52,73 años de edad, además detectaron ADN de VPH en 25 genotipos de 700 muestras con tumores de mama, lo cual representó un 3,57% del total. El VPH-16, fue el más concurrente, seguido por VPH-33 y VPH-18. En nuestro estudio, obtuvimos un 25% (8/32) de VPH-16 y 59,38% (19/32) de VPH-18. Esto nos indica que la prevalencia de infección del VPH varía entre las distintas zonas geográficas y estaría relacionado con factores no modificables como el factor genético y origen étnico, entre otros que dan lugar a la competencia del virus por nichos poblacionales con la prevalencia de un determinado tipo. Del total de genotipos encontrados, el 15,63% (5) correspondió a infecciones mixtas, relacionadas al grado II y al grado III del CaMa y a su vez al tipo de carcinoma ductal infiltrante. Este hecho nos permite deducir que además de la competencia viral entre los tipos de VPH en un mismo tejido, también se observa la persistencia de la infección y la progresión de las lesiones en la mama.

Con respecto a la edad, se observó que las edades de las mujeres positivas para VPH-16 y VPH-18 fueron diversas, por lo que no se pudo determinar la relación de edad y el tipo de VPH. Sin embargo, Kroupis *et al*. ^22^, luego de evaluar la presencia del VPH en casos de CaMa en 107 muestras, encontraron que 21 muestras fueron positivas para VPH de alto riesgo, siendo 14 de ellas más jóvenes que el resto de las pacientes. Por otro lado, Khan *et al*. ^8^ no encontraron significancia entre la edad y la presencia de VPH en CaMa, las edades de las pacientes fluctuaban entre 23 a 90 años y la mediana fue 55 años; de los 15 casos que eran menores de 40 años 3 fueron positivos para VPH, de los 37 casos que tenían edades entre 40 y 49 , 9 fueron positivos para VPH; de las 27 pacientes con edades entre 50 y 59 años, 5 albergaron el genoma viral del VPH y de los 45 casos que tenían edades entre 60 y 93, 9 fueron positivos para VPH. Por tanto, para poder entablar una posible relación de la edad y la presencia del VPH, se debe trabajar con un mayor número de muestras que permitan analizar la prevalencia de este virus. Los resultados de los estudios mencionados coinciden con el Plan Nacional para la Prevención y Control de Cáncer de Mama en el Perú 2017-2021, el cual reporta que el CaMa es más frecuente en mujeres peruanas de 40 a 69 años ^23^.

La aplicación de la IHQ en muestras de mama se realizó para determinar la sobreexpresión de los receptores C-erbB2 y Ki-67. Diversos estudios han utilizado estos marcadores y los han catalogado como específicos de células cancerosas de alta asociación con VPH de alto riesgo. A mayor expresión de Ki-67, mayor aumento de la expresión de C-erbB2 y, por lo tanto, mayor agresividad del cáncer ^16^. Esto explicaría la mayor sobreexpresión de C-erbB2 y Ki-67 en las muestras de CaMa de alto grado y sería el motivo por el cual se observó mayor sobreexpresión de estos receptores en el carcinoma ductal infiltrante. Por otro lado, las proteínas HER-2, se encuentran en la superficie de las células de la mama, tanto células normales como cancerosas ^17^. HER-2 se sobreexpresa hasta en un 30% en los cánceres de mama ^18^. La proteína Ki-67 se encuentra en el núcleo de la célula humana y expresa proliferación celular tanto en el tejido normal como en el maligno, alcanzando niveles máximos de expresión durante la mitosis ^19^. Actualmente, debido a que la característica principal del cáncer es la proliferación celular no controlada, se está usando más frecuentemente el índice proliferativo de Ki-67 para evaluar y controlar el cáncer como un indicador pronóstico ^18^ ^,^ ^19^. Al usar IHQ observamos la sobreexpresión de estos inmunomarcadores en las mujeres con CaMa positivas para VPH-18 en un 15,63% y para VPH-16% en un 6,25%. De las 19 muestras que dieron positivo a VPH-18 por qPCR, 5 muestras que sobreexpresaron los receptores ki-67 y C-erbB2 coincidieron con las dos técnicas; y de las 8 muestras que dieron positivo a VPH-16 por qPCR, 2 que sobre expresaron los receptores ki-67 y C-erbB2 coincidieron con las dos técnicas. Este hecho indicaría la alta precisión diagnóstica de la técnica qPCR, lo cual nos permitiría considerarla como una prueba complementaria a la IHQ, para posiblemente poder contribuir con el diagnóstico preliminar para el CaMa. 

Nuestros resultados concuerdan con lo descrito en el 2015 por Woods *et al*., quienes observaron que la coexpresión de E6 con C-erbB2 dio como resultado células que expresaban niveles más altos de C-erbB2, por lo que concluyeron que probablemente hay una influencia de la oncoproteína E6 del VPH-16 en la sobreexpresión del gen HER-2 (C-erbB2) ^20^. Sin embargo, hay estudios que indican lo contrario. En el 2015, Delgado analizó la relación entre el VPH y el marcador molecular Ki-67 en 275 muestras de cáncer de mama, sin encontrar significancia estadística entre la presencia de VPH y Ki-67 ^21^. No obstante, Kroupis *et al*. examinaron la presencia de VPH en tejidos de cáncer de mama por medio de la PCR, 17 muestras de 107 dieron positivo para VPH y encontraron significancia estadística entre VPH y Ki-67 ^22^. Por tanto, se concluye que, al trabajar con pocas muestras, no se puede determinar si la sobreexpresión de C-erbB2 y Ki-67 guarda o no relación con la presencia del VPH. 

Entre las limitaciones de este estudio debemos mencionar que no se determinó la prevalencia del VPH-16 y VPH-18 en el CaMa, y su correlación con los marcadores inmunohistoquímicos C-erbB2 y Ki-67. Por otra parte, entre las fortalezas, destacamos que se analizaron variables clínicas y sociales que no se han encontrado en otros estudios a nivel nacional. Asimismo, se tuvo en cuenta los diversos factores que influyen en la calidad del ADN, por ello se trabajó con un kit de extracción especializado para muestras de tejido mamario parafinado, se estandarizó la qPCR y se trabajó con amplicones menores a 200 bp, los cuales garantizan una alta precisión diagnóstica.

En conclusión, se evidenció la presencia de VPH-16 y VPH-18 en las biopsias de carcinoma mamario de pacientes peruanas, encontrándose mayor presencia de VPH en el carcinoma ductal infiltrante y de grado III. Asimismo, nuestros resultados nos han permitido confirmar que, además de la presencia del VPH, se requieren de otros factores intrínsecos o extrínsecos que actúan de forma sinérgica y predisponen el desarrollo del cáncer en el ser humano.
